# Implementation and Effects of an Information Technology–Based Intervention to Support Speech and Language Therapy Among Stroke Patients With Aphasia: Protocol for a Virtual Randomized Controlled Trial

**DOI:** 10.2196/30621

**Published:** 2021-07-02

**Authors:** Esther S Kim, Laura Laird, Carlee Wilson, Till Bieg, Philip Mildner, Sebastian Möller, Raimund Schatz, Stephanie Schwarz, Robert Spang, Jan-Niklas Voigt-Antons, Elizabeth Rochon

**Affiliations:** 1 Department of Communication Sciences and Disorders Faculty of Rehabilitation Medicine University of Alberta Edmonton, AB Canada; 2 Department of Speech-Language Pathology University of Toronto Toronto, ON Canada; 3 KITE Research Institute Toronto Rehabilitation Institute University Health Network Toronto, ON Canada; 4 Center for Technology Experience Austrian Institute of Technology Vienna Austria; 5 Nurogames GmbH Cologne Germany; 6 Quality and Usability Lab Technische Universität Berlin Berlin Germany; 7 German Research Center for Artificial Intelligence (Deutsches Forschungszentrum für Künstliche Intelligenz) Berlin Germany

**Keywords:** aphasia, rehabilitation, speech-language pathology, app-based therapy, user-centered design, mHealth, adaptive software

## Abstract

**Background:**

Mobile app–based therapies are increasingly being employed by speech-language pathologists in the rehabilitation of people with aphasia as adjuncts or substitutes for traditional in-person therapy approaches. These apps can increase the intensity of treatment and have resulted in meaningful outcomes across several domains.

**Objective:**

VoiceAdapt is a mobile therapy app designed with user and stakeholder feedback within a user-centered design framework. VoiceAdapt uses two evidence-based lexical retrieval treatments to help people with aphasia in improving their naming abilities through interactions with the app. The purpose of the randomized controlled trial (RCT) proposed here is to examine the feasibility and clinical efficacy of training with VoiceAdapt on the language and communication outcomes of people with aphasia.

**Methods:**

A multicenter RCT is being conducted at two locations within Canada. A total of 80 people with aphasia will be recruited to participate in a two-arm, waitlist-controlled, crossover group RCT. After baseline assessment, participants will be randomized into an intervention group or a waitlist control group. The intervention group participants will engage in 5 weeks of training with the app, followed by posttreatment and follow-up assessments after an additional 5 weeks. Those in the waitlist control group will have no training for 5 weeks; this is followed by pretreatment assessment, training for 5 weeks, and posttreatment assessment. All trial procedures are being conducted remotely given the COVID-19 pandemic.

**Results:**

Recruitment of participants started in September 2020, and the study is expected to be completed by March 2022. Publication of results is expected within 6 months of study completion.

**Conclusions:**

The results of the RCT will provide information on evidence-based practice using technology-based solutions to treat aphasia. If positive results are obtained from this RCT, the VoiceAdapt app can be recommended as an efficacious means of improving lexical retrieval and communicative functioning in people with aphasia in an easily accessible and a cost-effective manner. Moreover, the implementation of this RCT through remote assessment and delivery can provide information to therapists on telerehabilitation practices and monitoring of app-based home therapy programs.

**Trial Registration:**

ClinicalTrials.gov NCT04108364; https://clinicaltrials.gov/ct2/show/NCT04108364

**International Registered Report Identifier (IRRID):**

DERR1-10.2196/30621

## Introduction

### Background

Aphasia is an acquired language disorder, most commonly caused by stroke. It can impact linguistic expression and comprehension in written and oral modalities. As such, this condition can have devastating consequences on the social, emotional, and occupational functioning of people with aphasia [1]. Although aphasia affects nearly one-third of all individuals who experience a stroke [2,3] and has been associated with poorer outcomes in the acute and chronic periods following stroke [4], the condition itself remains relatively unknown among the general public [5].

Rehabilitation for people with aphasia provided by speech-language pathologists (SLPs) has shown improved outcomes [6,7]. However, several factors, including constraints on health care resources, often prevent people with aphasia from receiving services, particularly in the chronic stages. The recent proliferation of mobile app–based therapies (apps) has provided a cost-effective means by which people with aphasia can receive prolonged rehabilitation. SLPs have been increasingly employing such apps as adjuncts or substitutes for traditional (in-person) therapy approaches, with meaningful outcomes observed across several domains [8,9]. Such apps have the potential to increase the intensity of rehabilitation, a factor that is associated with greater long-term recovery [10-12]. Moreover, recent evidence has shown that people with aphasia can self-deliver this type of intervention and attain positive language and communication outcomes [13-15]. Technology also enables measuring multiple parameters of user interactions with touch-based devices, and algorithms can be deployed to adapt content based on the user/patient state.

Large-scale randomized controlled trials (RCTs) investigating the clinical utility of tablet-based speech-language therapy apps for aphasia have not been conducted. Nevertheless, preliminary evidence on the utility of app-based therapy for adults with acquired aphasia is emerging. Mallet and colleagues [16] demonstrated that mobile tablet–based rehabilitation is feasible for delivering speech-language therapy in an acute care setting. In this study, patients with communication deficits following acute stroke interacted with tablets containing commercially available speech-language therapy apps. The participants in this study expressed a desire to be more active in their rehabilitation, and they even exceeded the minimum time (one hour per day) recommended for engaging with the mobile tablet. In a more recent RCT conducted on 32 people with aphasia, Braley and colleagues [13] reported that people with aphasia in the treatment group who practiced a variety of speech, language, and cognition exercises using a tablet-based therapy app (ie, Constant Therapy) demonstrated greater language outcomes than a group who completed worksheet exercises. Importantly, this RCT was conducted virtually with participants self-managing their participation through periodic monitoring by the researchers. Additional evidence derived from case series and small group studies also proves the utility of app-based therapy for adults with aphasia in the chronic stages. Participants in these studies have shown improvements across a variety of outcome measures, including naming of trained and untrained items, standardized language and cognitive assessment batteries, and spoken discourse [15,17-19].

Despite the increasing availability of therapy apps designed for aphasia [20], very few have been designed with inputs from relevant stakeholders following the principles of user-centered design (UCD). UCD is a systematic approach to meet usability and user experience goals through integrating the needs and abilities of users during the design process of an artifact. This ensures that the product/service design and development align with all the relevant stakeholders’ needs (ISO 9241-220). Essential UCD activities include planning and managing the process, defining the context of use for each user group, gathering user and stakeholder requirements, designing an ergonomic solution based on the requirements, and evaluating the design within a user-centered approach [21].

In a recent review, only three apps were identified where developers engaged people with aphasia in the design process [22]. All these apps engaged patients to varying extents during therapy evaluation or prototype testing. There was no evidence of patients being involved in either of the two initial phases of app development (ie, theory-based conception, software implementation). Based on the above review, the authors present a broad, four-phase model of the app development process encompassing the following: (1) theoretical considerations, (2) software development, (3) pilot testing, and (4) long-term evaluation. The authors conclude that people with aphasia should be involved in all phases during the development and evaluation of therapy apps, as their involvement could “enhance the product quality, functionality and acceptability, and increase patients’ motivation to use these digital therapies” [22].

### VoiceAdapt

Recently, the VoiceAdapt Consortium (VC), an international team of researchers from the fields of computer science, usability design, and speech-language pathology, has produced an app for treating naming impairments in people with aphasia. Using evidence-based lexical retrieval protocols, the VoiceAdapt app instantiates Phonological Components Analysis (PCA) [23] and Semantic Feature Analysis (SFA) [24] into an adaptive, voice-responsive therapy app that people with aphasia can use to improve their naming skills.

The VoiceAdapt project has so far involved 75 end users (people with aphasia, clinicians, and caregivers) in the UCD process to identify the main barriers and obstacles to app-based training and rehabilitation; ensure independent usage and sustained motivation; and optimize the end-user experience and training impact. This was achieved by eliciting user requirements (conducting guideline-based structured interviews), designing solutions (mock-ups, prototypes), and evaluating the designs against the requirements (focusing on user experience in terms of multimodal speech interaction and adaptivity) over three iterative cycles. The result is a mobile speech-language training app for people with aphasia that can run on iOS and Android platforms. The app supports regular training sessions, adaptivity (individual performance thresholds for training material selection), personalization (training content is selected based on individual interests), and coordination between therapists and patients; it also addresses multimodal language goals (reading, understanding, and production).

### Study Purpose

The purpose of this study is to examine the impact of the VoiceAdapt adaptive speech-language treatment app, created using the principles of UCD, on the naming abilities of people with aphasia within an RCT. Secondary outcomes focusing on overall language improvement, communication, and quality of life will also be examined. Herein, we present the protocol of the VoiceAdapt study (ClinicalTrials.gov NCT04108364).

## Methods

### Selection of Communities for the Study, Participants, and Procedures

Participants will be recruited from two geographic regions in Canada through the Language Sciences Lab (Department of Speech-Language Pathology, University of Toronto) and Aphasia Research Lab (Department of Communication Sciences and Disorders, University of Alberta), based in Toronto, Ontario and Edmonton, Alberta, respectively. Participants with aphasia will be recruited from community-based aphasia centers and programs, clinics, and research databases. Initial contacts will be made with aphasia centers and programs providing information about the study, followed by recruitment presentations and meetings with interested participants.

### Inclusion and Exclusion Criteria

The participants enrolling in the study will meet the following inclusion criteria: be at least 6 months post the onset of a stroke in the left hemisphere; have mild-to-moderate aphasia (Aphasia Quotient ≥30 on the Western Aphasia Battery-Revised [WAB-R]) [25]; score below 75% correct on the Boston Naming Test (BNT) [26] during initial assessment; exhibit prominent verbal expression impairment; pass screenings for vision, hearing, and basic cognitive functioning; speak English as a primary language; and be willing to commit to participation for the entire study duration. Despite meeting all these inclusion criteria, participants will be excluded if they are currently engaged in individual (one-on-one) speech-language therapy or are using speech-language therapy apps.

### Study Design

**Figure 1:**
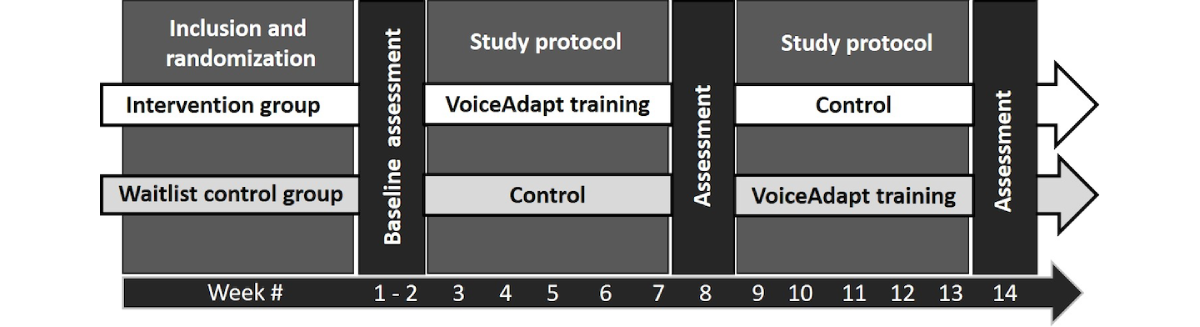
Schematic of the study design.

The RCT will have a two-arm, randomized, waitlist-controlled, crossover group design. In Phase 1, people with aphasia randomized to the intervention group will train with VoiceAdapt. The control group will be a waitlist treatment-deferred group. In Phase 2, the control group will train with VoiceAdapt (see Figure 1). Participants will be randomized to groups according to a computer-generated randomization list.

### Intervention

**Figure 2:**
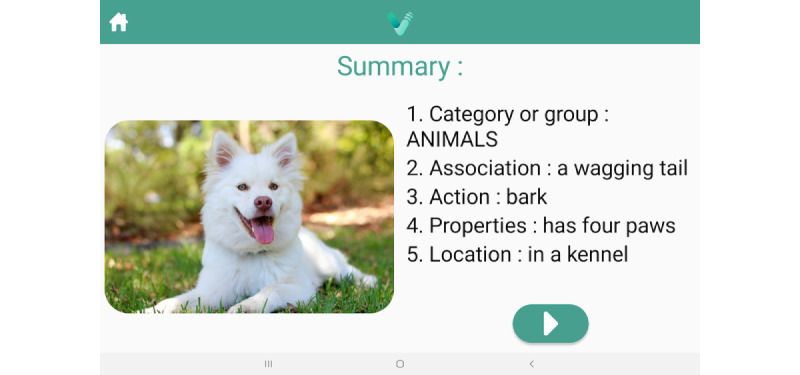
Screenshot from the VoiceAdapt app displaying the summary screen of the Semantic Feature Analysis protocol.

**Figure 3:**
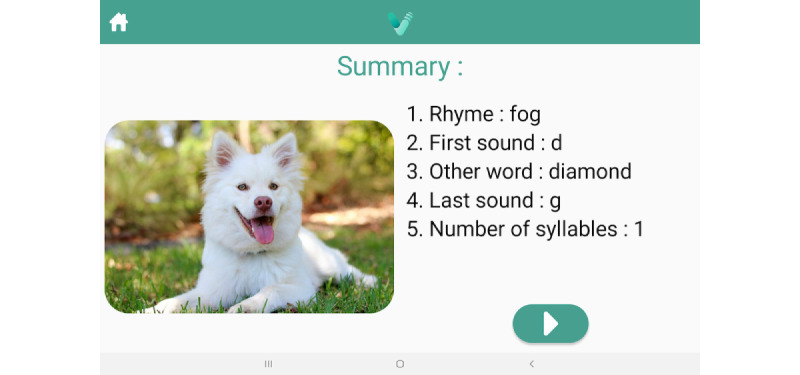
Screenshot from the VoiceAdapt app displaying the summary screen of the Phonological Components Analysis protocol.

**Figure 4:**
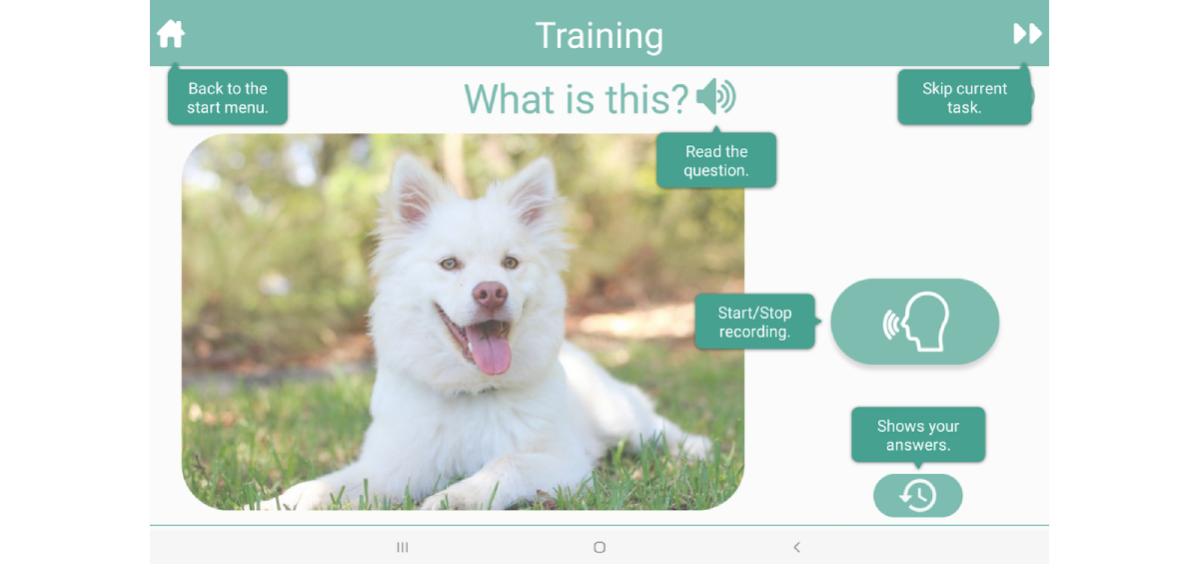
Screenshot from the VoiceAdapt app displaying the user help screen.

The app intervention comprises naming exercises based on evidence-based anomia treatments, SFA and PCA, delivered on a mobile tablet. The exercises involve the presentation of a colored picture and a series of prompts designed to engage the people with aphasia in providing semantic features (eg, “What is it used for?” [action]; “What does it look like?” [properties]); or phonological components (eg, “What sound does it start with?” [first sound]; “What is a rhyming word?” [rhyme]). Figures 2 and 3 display summary screens reviewing the generated semantic and phonologic features of the target items, respectively (Additional screenshots of the app are displayed in Multimedia Appendix 1). A help screen also reminds users of the various features (Figure 4). The patient’s voice is recorded and used as the input for the app. The app is designed to maximize user engagement through UCD principles. People with aphasia randomized to the VoiceAdapt treatment group will be instructed to practice using the app during weekdays for one hour per day for 5 weeks. The people with aphasia will have the option of downloading the app on their own tablets from a link provided by the researchers, or the researchers will provide them with tablets with the app loaded. Research staff will check in and monitor progress through email or telephone/video calls on a weekly basis.

### Duration

The participants will take part in the study for approximately 13 weeks once enrolled. Pretreatment assessment will take place in Week 1, followed by 5 weeks of engagement in Phase 1 (treatment or control). Midpoint assessments will take place in Week 7, followed by 5 weeks of engagement in Phase 2 (crossover into control or treatment). Final assessments will take place in Week 13 following study enrollment.

### Measures

Efficacy will be monitored by the scores on primary and secondary outcome measures. The primary outcome measure will be the BNT [25] to measure naming; secondary outcome measures will be the WAB-R [26]; Stroke and Aphasia Quality of Life Scale-39 (SAQOL-39) [27] to measure quality of life; and Communication Effectiveness Index (CETI) [28] to measure the communication capabilities of the participants with aphasia and their partners’ perception of the participants’ communication. In addition to the primary and secondary outcome measures, the System Usability Scale (SUS) [29], Situational Motivation Scale (SIMS) [30], and a posttreatment questionnaire specific to app usage will be administered to all participants. In addition, the in-app parameters evaluating aspects such as the amount of time spent on training per session, total amount of time spent on training over the study duration, etc will also be measured for future analysis.

### Sample Size Determination

The planned enrollment for the study is 80 (ie, 40 at each of the two sites in Canada). The sample size calculation is based on the medium effect sizes estimated in our study according to previous results. Accounting for a dropout rate of approximately 10%, we need to include 79 people with aphasia; thus, we plan to enroll 80 people with aphasia. We will conduct an intention to treat analysis and include all the randomized participants in our analyses.

### Analytic Strategy

Mixed-effects regression will be used to analyze the 2 × 2 crossover trial with one baseline [31]. The analysis will include covariate adjustments for variables such as the recruitment site and participant characteristics (eg, sex, age, hours of training, assessors, and caregiver characteristics). Adjustment can increase the precision of the treatment effect estimates and confirm whether training was equally efficacious across covariates (ie, using covariates by treatment interaction) and whether the retention of improvement was equal across covariates (ie, covariates by carryover interaction). Mixed-effects regression can also account for hierarchical sampling, which is important if participants within sites are more similar than if they were randomly chosen. If carryover is detected, we will also estimate the treatment effects after excluding the Period 2 assessments affected by carryover.

The Mauchly test will be used to evaluate whether the sphericity assumption is tenable. If the variances are unequal, we will disaggregate the analysis and use paired and two-group *t* tests as appropriate to estimate the treatment, carryover, and period effects; if covariate adjustment is required, these contrasts will be evaluated using mixed-effects regression while also accounting for hierarchical sampling.

Interim analyses will be conducted when quarterly enrollment targets are met (ie, 20, 40, and 60 participants).

### Ethics Statement and Consent

This study will be conducted according to Canadian and international standards of Good Clinical Practice for all studies. Applicable government regulations and the University of Toronto and University of Alberta research policies and procedures will also be followed.

This protocol and any amendments to the same will be submitted to the University of Toronto Human Research Ethics Unit (HREU) and University of Alberta Health Research Ethics Board (HREB) for formal approval to conduct the study.

All participants selected for this study will be provided a consent form describing the study and providing sufficient information for them to make an informed decision about their participation in this study. This consent form will be submitted with the protocol for review and approval by the HREU and HREB. The formal consent of a participant, using the HREU- and HREB-approved consent form, will be verbally obtained and recorded before that participant is subjected to any study procedure per HREU- and HREB-approved procedures.

### Adaptations for Data Collection During the COVID-19 Pandemic

With in-person research activities limited due to the COVID-19 pandemic, all data collection activities will be conducted virtually using HREU- and HREB-approved video conferencing software. Recruitment presentations, initial informational/screening interviews, and all assessment sessions will take place remotely. All assessment instruments have been adapted for remote delivery and will be administered by the assessors through sharing their screen and eliciting verbal responses or pointing to responses using annotation functions within the software. All adaptations to the research protocol for remote delivery have been approved by the Research Ethics Boards of the University of Toronto and University of Alberta.

## Results

The project was funded in 2018 and enrolment for the RCT is ongoing. A primary completion date is expected in March 2022, and publication of results is expected within 6 months of work completion.

## Discussion

This RCT is designed to assess the efficacy of the VoiceAdapt app, a new, tablet-based therapy program for people with aphasia. This app was designed using the principles of UCD, incorporating inputs from the end users (people with aphasia, their caregivers, and SLPs) through an iterative process. The end result is a mobile speech-language training app instantiating evidence-based treatment principles into an adaptive and a personalizable app that addresses multimodal language stimulation. The RCT will examine the effect after 5 weeks (25 hours) of training with the app on language production (ie, naming) in people with aphasia in the chronic stage of recovery. The impact on secondary outcomes, including communication effectiveness and quality of life, will also be assessed.

Technology-based solutions, including apps to reach speech-language therapy and communication goals can be a means to increase home practice and therapy intensity in a cost-effective manner. Particularly for people with aphasia who are in the chronic stages of recovery (ie, more than a year post the onset of aphasia), there can be limited rehabilitation options. It is recommended that technology-based rehabilitation approaches engage participants in a personalized manner [8,13]. Although an increasing number of therapy apps are entering the market, they do not engage people with aphasia in the development and testing stages [22]. The VoiceAdapt app differs from other apps currently available on the market in that it incorporates evidence-based treatment approaches within a framework that was identified by the end users through a UCD process in the conceptualization, development, and implementation stages of the app.

If positive results are obtained from this RCT, the VoiceAdapt app can be recommended as an efficacious means of improving lexical retrieval and communicative functioning in people with aphasia in an easily accessible and a cost-effective manner. Moreover, the implementation of this RCT through remote assessment and delivery can provide information to therapists on telerehabilitation practices and monitoring of app-based home therapy programs.

## Appendix

Multimedia Appendix 1 Screenshots of VoiceAdapt app.

Multimedia Appendix 2 Peer-review report by the funding committee of the JTC 2017 launched by the Joint Programming Initiative “More Years Better Lives” (JPI MYBL).

## Abbreviations

BNT: Boston Naming Test
CETI: Communicative Effectiveness Index
HREB: Health Research Ethics Board
HREU: Human Research Ethics Unit
RCT: randomized controlled trial
PCA: Phonological Components Analysis
SAQOL-39: Stroke and Aphasia Quality of Life Scale - 39
SFA: Semantic Features Analysis
SLP: speech-language pathologist
SIMS: Situational Motivation Scale
SUS: System Usability Scale
UCD: user-centered design
WAB-R: Western Aphasia Battery-Revised
