# Supplementary material for: Implementation and Effects of an Information Technology–Based Intervention to Support Speech and Language Therapy Among Stroke Patients With Aphasia: Protocol for a Virtual Randomized Controlled Trial
Source: JMIR Res Protoc. 2021 Jul 2;10(7):e30621. doi: 10.2196/30621 (PMC8285741; doi:10.2196/30621)

Implementation and Effects of an Information Technology-Based Intervention to Support Speech and Language Therapy Among Stroke Patients with Aphasia: Protocol for a Virtual Randomized Controlled Trial

VoiceAdapt App Screenshots

Esther S. Kim, PhD, Laura Laird, BA, Carlee Wilson, MSc, Till Bieg, MSc, Philip Mildner, PhD, Sebastian Möller, PhD, Raimund Schatz, PhD, Mag. Stephanie Schwarz, Robert Spang, MSc, Jan-Niklas Voigt-Antons, PhD & Elizabeth Rochon, PhD

Login page

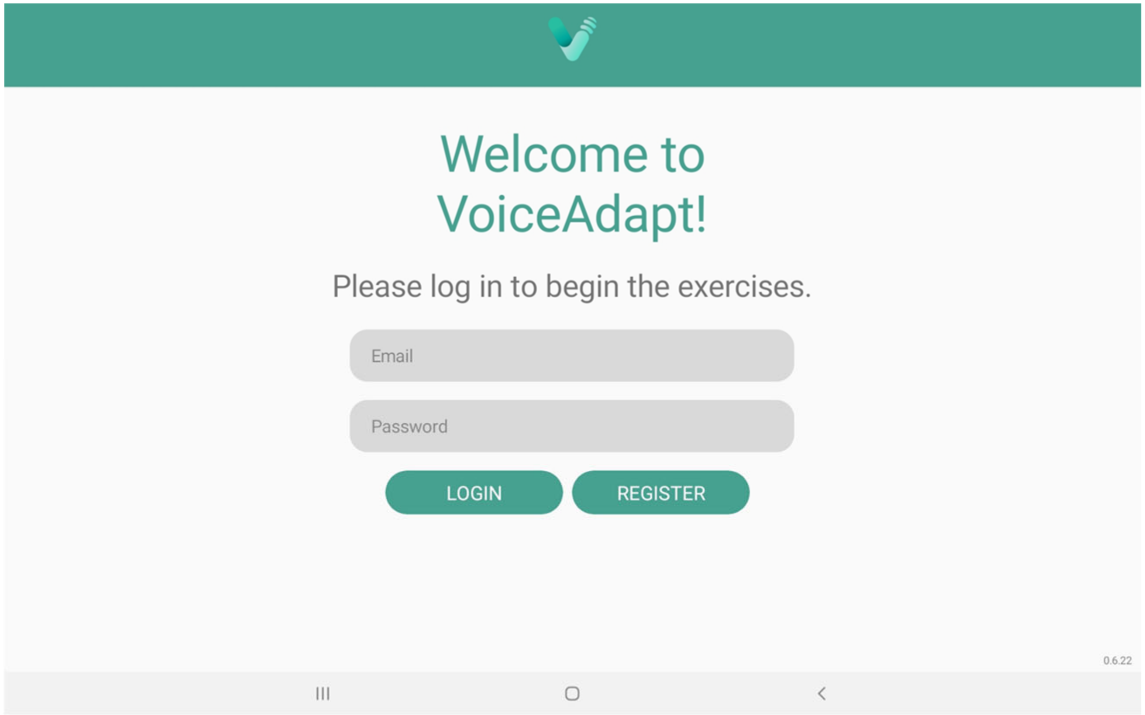
Welcome to
VoiceAdapt!
Please log in to begin the exercises.
Email
Password
LOGIN
REGISTER
0.6.22

Registration:
User profile

Here the user fills out profile information that is used to generate training content personalized to their interests.

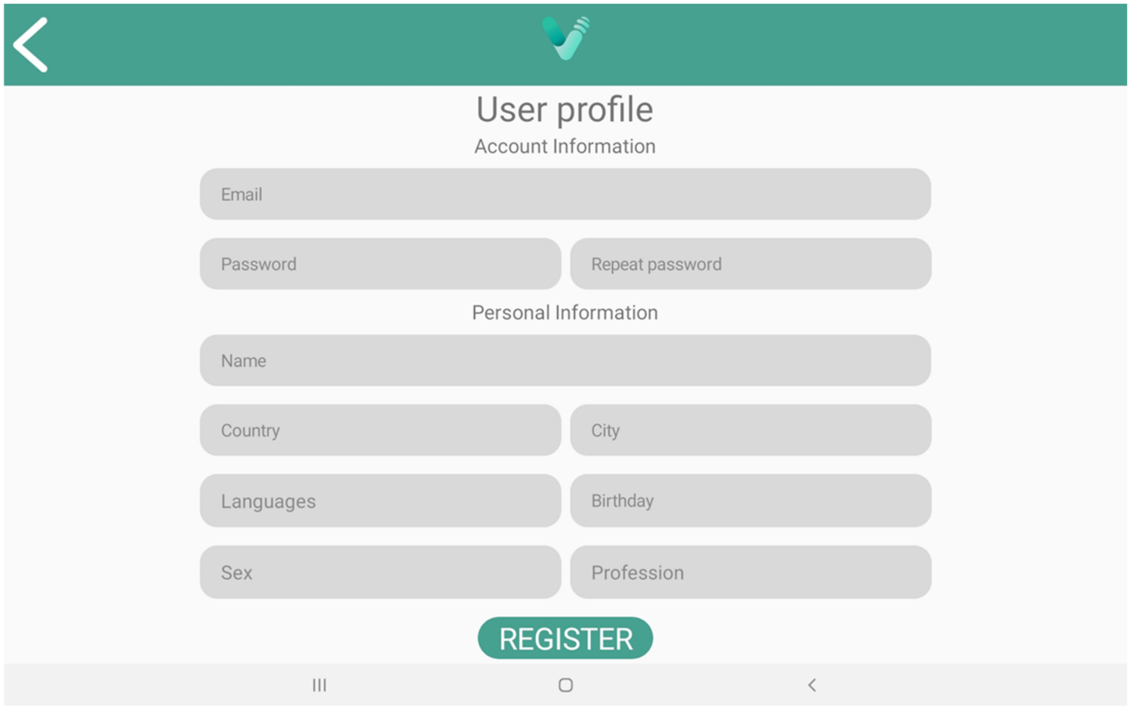

Start page

The therapist sets the weekly goal (60 words/week for participants enrolled in the trial). The progress bar shows weekly progress.

The app alternates between training items using Semantic Feature Analysis (SFA) and Phonological Components Analysis (PCA) each week.

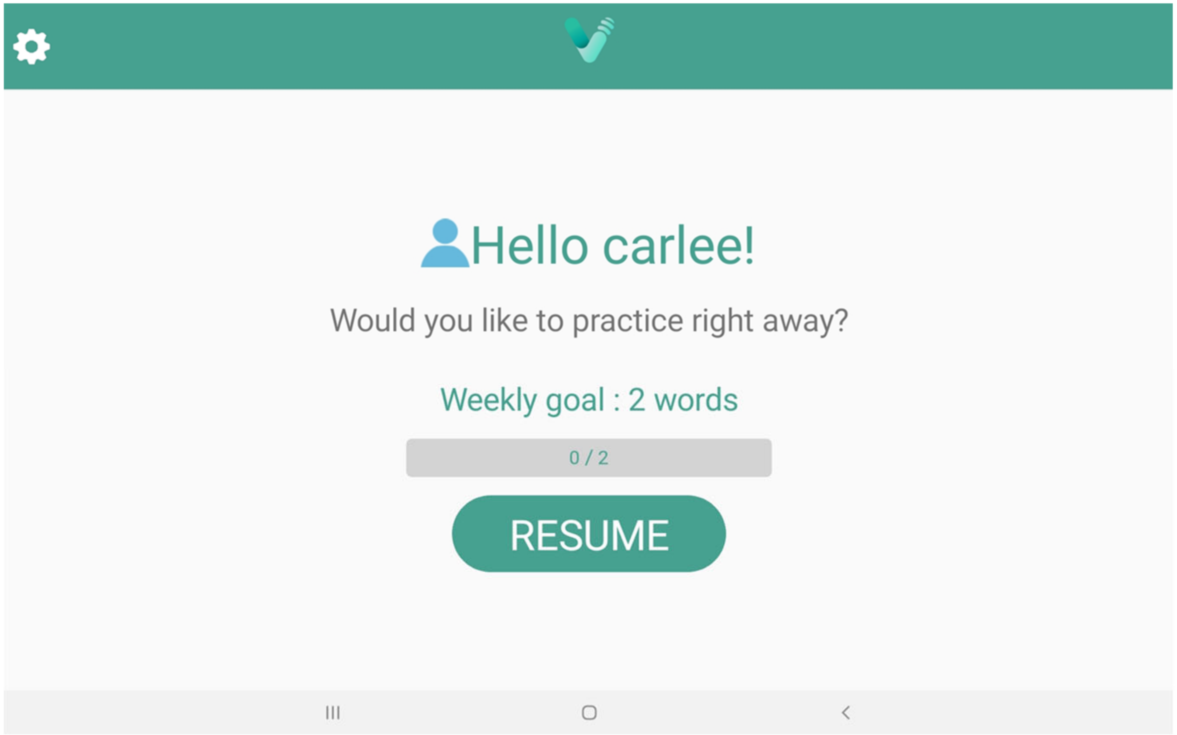

Naming Probe

The user is first presented with the target item and asked to name it.

The naming probe is presented two more times for each item: 1) following presentation of all of the semantic feature or phonological component cues; 2) following a summary review of cues presented.

The red ‘wave’ icon indicates that the app is recording the participant’s response.

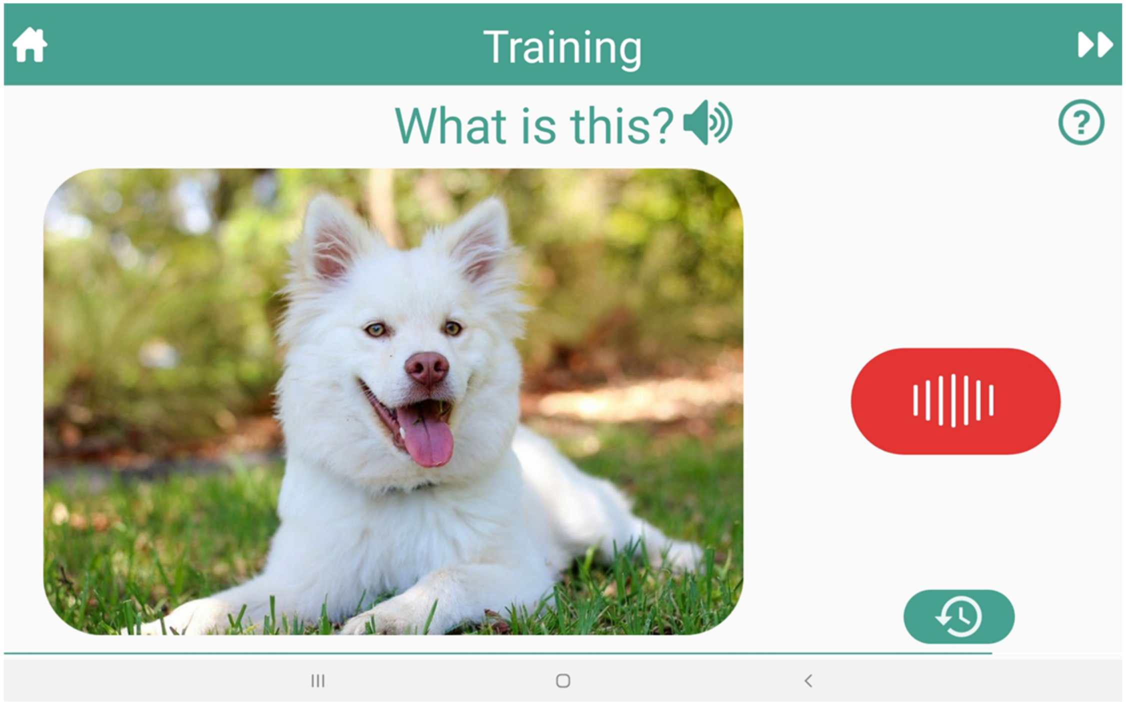

Semantic Feature Analysis (SFA)

Following the initial naming probe, the app presents the following cues:

- *Category or group*: “What category or group does this item belong to?”
- *Association:* “What does this make you think of?”
- *Action:* “What is it used for?”
- *Properties:* “What properties does the item have?”
- *Location:* “Where can you find the item?”

A second probe (“Now try to name the picture again. What is this?”) is presented, followed by a Summary page reviewing all of the cues and one final probe (“Name the picture one last time.”)

The following slide shows a screenshot example of one of the SFA prompts presented to users of the app.

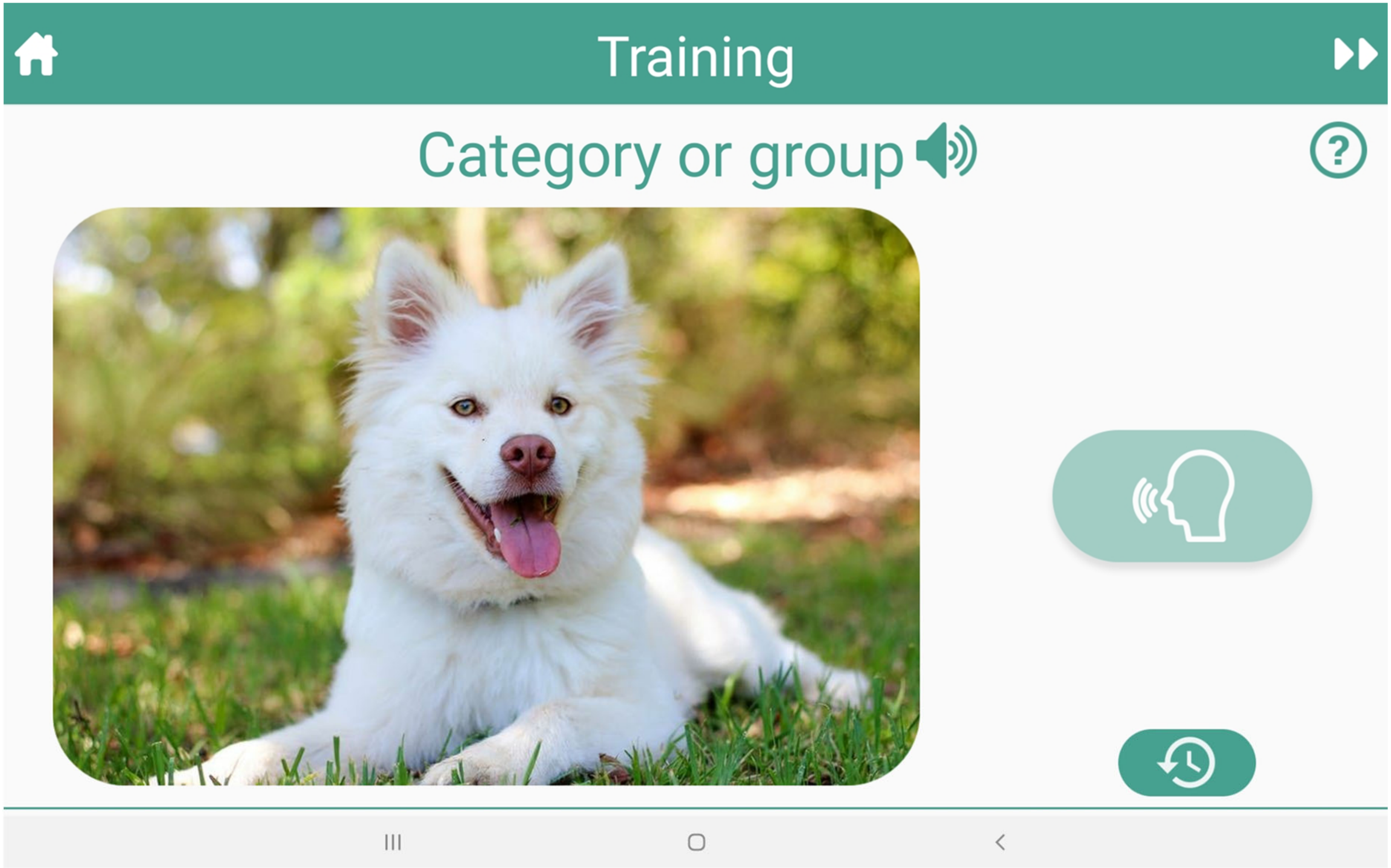
Training
Category or group

Summary :

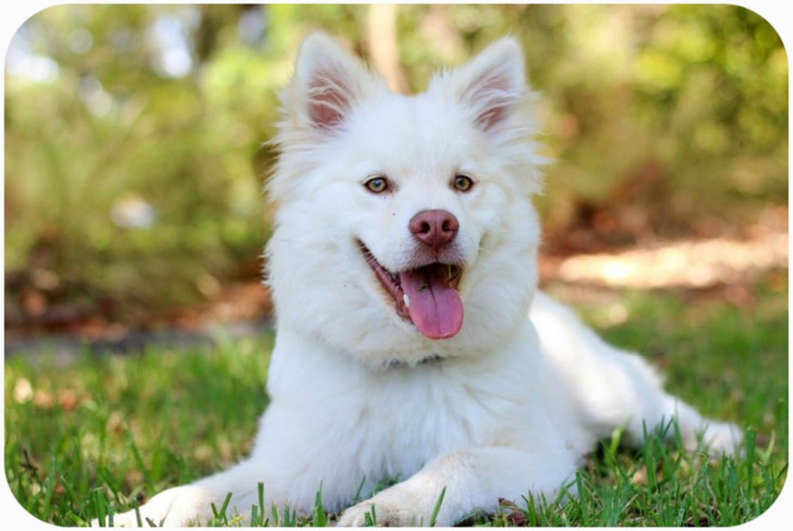

1. Category or group : ANIMALS
2. Association : a wagging tail
3. Action : bark
4. Properties : has four paws
5. Location : in a kennel

Phonological Components Analysis (PCA)

Following the initial naming probe, the app presents the following cues:

- *Rhyme:* “What rhymes with the word?”
- *First sound:* “What sound does the word in the picture start with?”
- *Other word:* “What is another word with the same first sound?”
- *Last sound:* “What is the last sound in this word?”
- *Number of syllables:* “How many syllables does the word have?”

A second probe (“Now try to name the picture again. What is this?”) is presented, followed by a Summary page reviewing all of the cues and one final probe (“Name the picture one last time.”)

The following slide shows a screenshot example of one of the PCA prompts presented to users of the app.

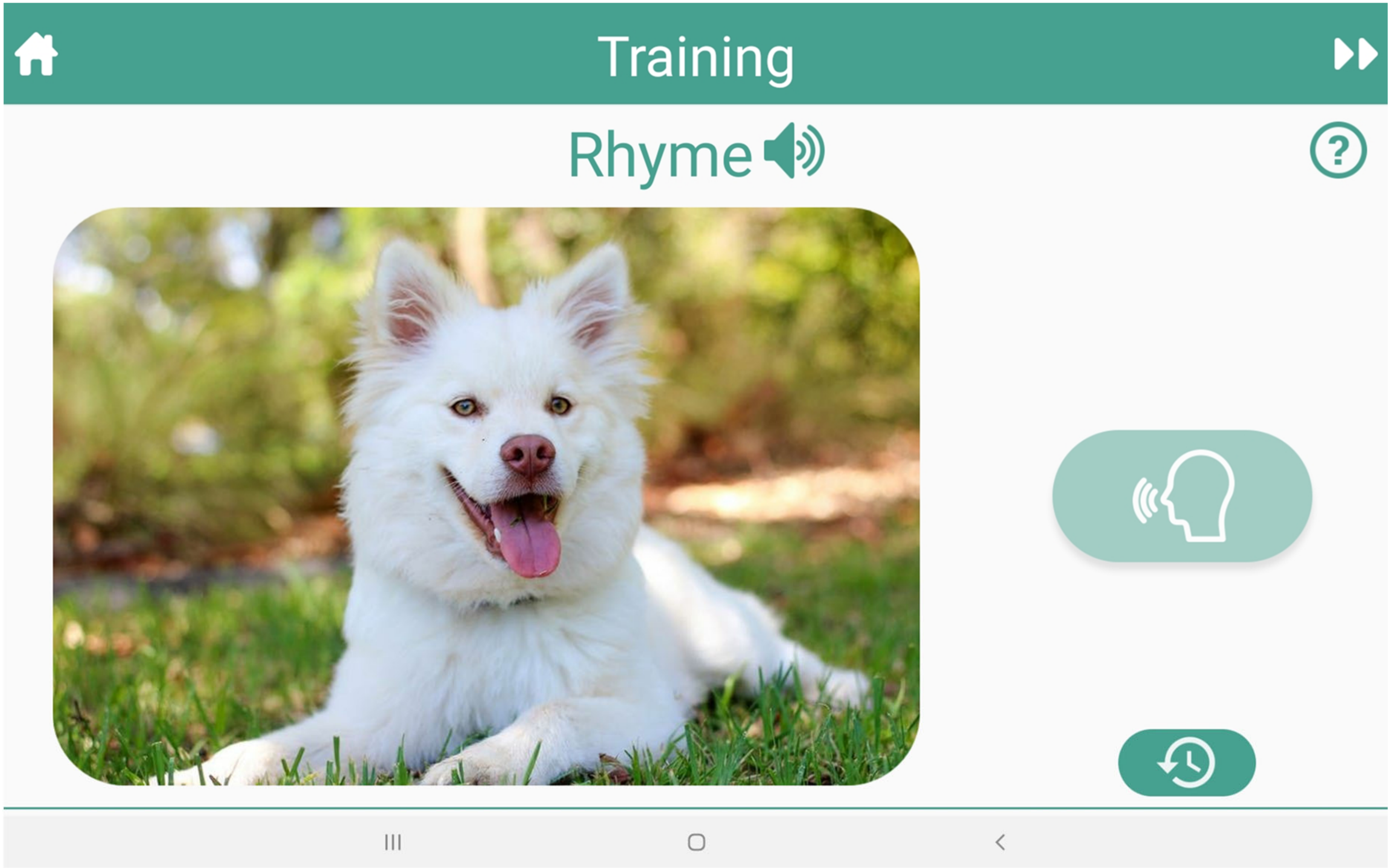
Training
Rhyme

Summary :

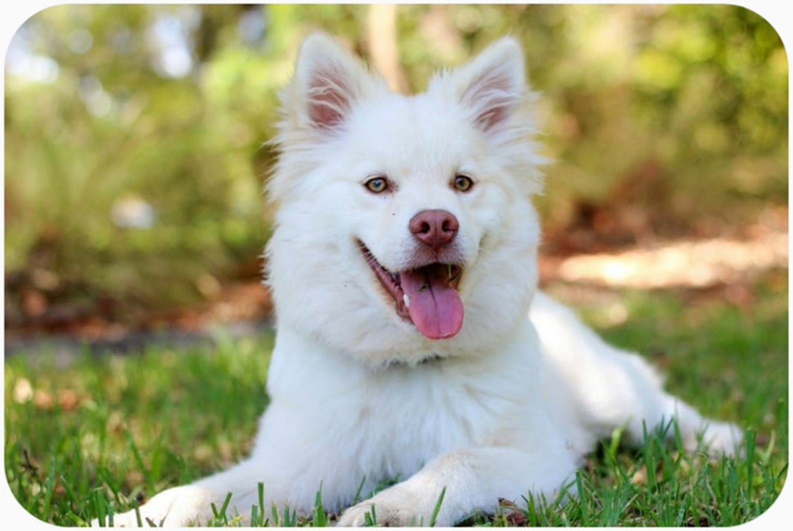

1. Rhyme : fog
2. First sound : d
3. Other word : diamond
4. Last sound : g
5. Number of syllables : 1

Final Screen

The user is presented with a final feedback screen before progressing to the next item.

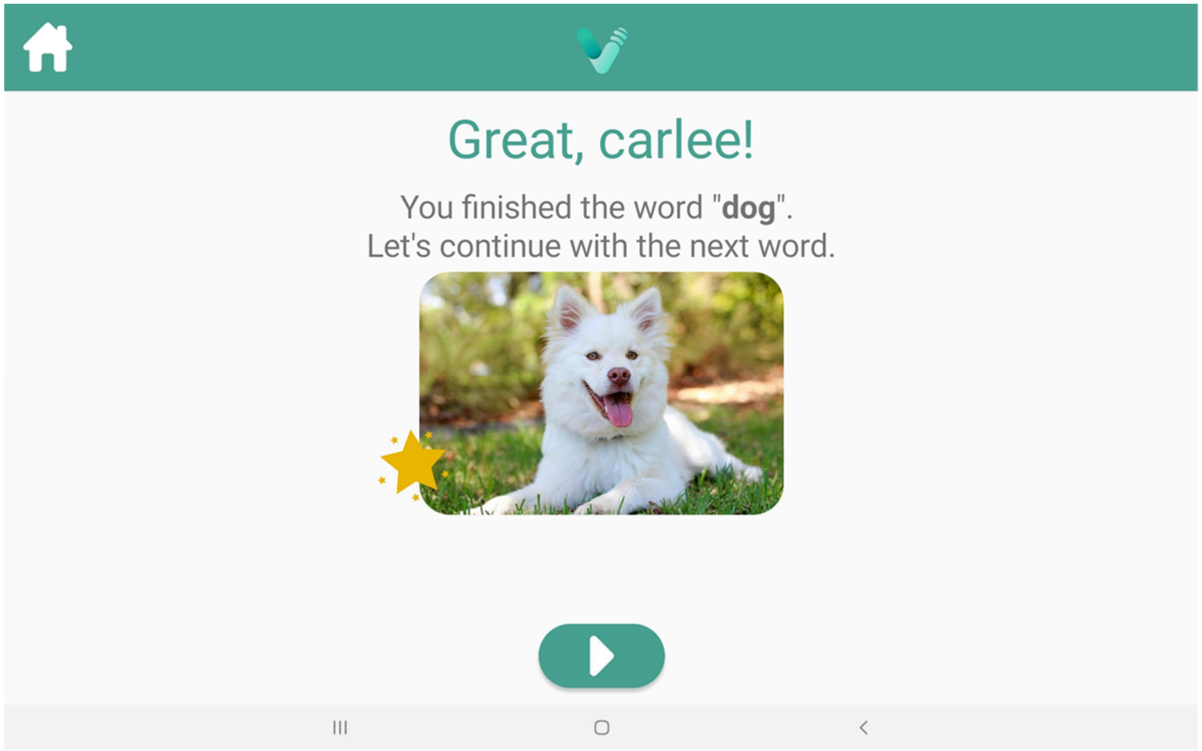

Supplement: Multimedia Appendix 1 [file resprot_v10i7e30621_app1.pdf]
