# Supplementary material for: Implementation and Effects of an Information Technology–Based Intervention to Support Speech and Language Therapy Among Stroke Patients With Aphasia: Protocol for a Virtual Randomized Controlled Trial
Source: JMIR Res Protoc. 2021 Jul 2;10(7):e30621. doi: 10.2196/30621 (PMC8285741; doi:10.2196/30621)
Supplement: Multimedia Appendix 2 [file resprot_v10i7e30621_app2.pdf]

# Proposal n° jpi03-094: VoiceAdapt

## *Voice Adaptive Training for older adults with Aphasia*

## Overall Evaluation

| Evaluators | Overall score (addition of scores 1 to 5) | Arithmetic mean |
|---|---|---|
| **Evaluator 1** | 25 | 25,33 |
| **Evaluator 2** | 29 | |
| **Evaluator 3** | 22 | |

| | Overall comments |
|---|---|
| **Rapporteur** | VoiceAdapt is recommended for funding. The proposal presents a clear and well detailed proposition to empower older adults with aphasia by providing them with an autonomously usable, low cost speech and language solution. Only several minor issues of concern are noted, primarily; The personnel and overhead budget for Canadian partners needs further clarification and further details should be provided to how outputs from the Canadian RCT's will impact stroke patients in the EU. |

## Evaluation Criteria

| **1. Relevance** | Clarity of the objectives and their respective relevance in relation to the aims of the call | **Score** |
|---|---|---|
| **Evaluator 1** | The proposal presents relevance for the need to enhance the capabilities of digital health to support the training (via speech interaction and context-aware driven lessons) of older adults living with aphasia at a low cost.<br><br>The project back ground, problem and objectives are clearly stated. Moving training for individuals with aphasia from a passive educational experience to a more proactive learning environment, tailored to their language and cognitive capabilities is an exciting prospect. | 4 |
| **Evaluator 2** | VoiceAdapt seeks to develop innovative speech recognition and adaptive learning solutions to support sufferers of Aphasia, a condition associated with stroke patients. The proposal objectives are relevant to the aims of the call although addressing a subset of the elderly population. The methodology and principles behind VoiceAdapt could be applied to other groups within the older population. | 5 |
| **Evaluator 3** | Interesting and relevant proposal with a good potetial to have an impact. | 4 |

| **2. Scientific Quality** | Scientific excellence of the proposal in terms of innovative approach, originality and expected progress beyond the state of the art, availability and quality of existing data, comparative perspective and interdisciplinarity. | **Score** |
|---|---|---|
| **Evaluator 1** | Key to driving the state of the art behind this proposal is the use of predictive learning to build on the user's existing speech level and personalise the training needed as part of the 'speech and language' rehabilitation process. The project ambitiously states that it's personalisation engine can dynamically direct multimodal learning content to the user based on voice | 4 |

| | | |
|---|---|---|
| | and interaction pattern analysis recognising the mental and cognitive capabilities of the user. This is both a highly innovative and ambitious proposal that clearly shows how the research will build on the current state of the art covering 3 core areas; Adaptation and personalisation; User and context modelling; Gamification and persuasion.<br>The geo-tag learning feature outlined on p.8 as part of the user scenario (Frank) presents a positive vision for context driven, ageing in place learning for individuals with aphasia.<br>Gamification within the proposal is mentioned on several occasions, however more specifics to its implementation (or methodologies to be used) would enhance understanding of the consortiums approach to this area.<br>Co-design methods with a good overview of the stakeholder panel to inform this approach is presented.<br>End user outcome measures are clearly listed on p.8. These outcomes primarily focus on health, communication and well-being. A list of measures/approaches to evaluate issues including user engagement, accessibility, acceptable and behavioural change would improve the proposal.<br>Sample size is well documented, however issues around proposed recruitment of participants needs further clarification.<br>A final issue in need of clarification is whether only individuals who have prior access to a suitable tablet meet the inclusion criteria for the RCT? If so does this limit the study to lead users and exclude a subset of individuals who may benefit substantially from an introduction to digital training/rehabilitation programmes. | |
| **Evaluator 2** | The proposal is well constructed and innovative and should contribute progress beyond the state of the art in the area of technology solutions based on the self-management of chronic conditions. The consortium team has strong interdisciplinarity with representation from both technology, medicine and behavioural sciences. | 5 |
| **Evaluator 3** | The team behind voiceAdapt pan to conduct RCT . will demonstrate the value of the tool | 3 |

| **3. Quality of the Project Consortium** | International competitiveness of participants in the field(s), previous work and expertise of the participants, added value of the transnational collaboration, participation of junior researchers | **Score** |
|---|---|---|
| **Evaluator 1** | The project presents an experienced team between Germany, Austria and Canada, highlighting strong international collaboration between partnes. The structure of the international collaboration presents a positive framework for the successful execution of the research programme. However some further clarity is needed with regards how technical assistance for the RCTs will be provided from the EU member states to the Canadian partners. Also the proposal would benefit from an increased understanding of how outcomes will be exploited/translated in the EU context particularly in terms of rehabilitation medicine post stroke.<br>The additional projects the consortium is engaged with presents significant added value to the knowledge base for implementing the VoiceAdapt programme as well as providing a cross translational research platform to other related conditions under examination in these projects. | 5 |
| **Evaluator 2** | The consortium has representation from both Europe and Canada with a very strong team of experienced personnel and planned involvement of junior researchers. The ongoing commercial sustainability of the proposal is also covered | 5 |

| | | |
|---|---|---|
| **Evaluator 3** | Good and relevant | 4 |

| **4. Feasibility of project plan** | Relation of work packages to proposal themes and aims, quality of work plan and time schedule, balanced participation of project partners, quality and efficiency of the coordination and management, scientific justification and adequateness of the requested budget and risk assessment | **Score** |
|---|---|---|
| **Evaluator 1** | WPs are well structured and clearly presented. A minor point of note in WP2 is the substantial PM contribution placed on AIT, as evaluations are anticipated equally in two separate locations. Will AIT be primarily responsible for the evaluation? This needs clarified.<br><br>The project coordination, organisation and management is clearly explained and suitable to the programme.<br><br>Gantt chart is clearly presented. A few minor points in need of clarification are:<br>- T2.4 ends after T3.4: Should the development not end after the user experiences have been appropriately evaluated with outputs transferred into the service interaction and design?<br>- T5.1 - The project website development is complete in M4 with no further updates indicated across the lifetime of the project.<br>- D5.2 – It would be recommended to have a mid point review of the plan/report.<br><br>Project ethical and IP requirements are acceptably addressed.<br><br>The budgets for EU partners is well outlined. Some queries are present for the Canadian partners.<br>• Firstly the personnel costs seem low for these teams running the RCT's. This should be clarified. Given the extensiveness of the research it is not clear whether these low personnel costs mean that the graduate students listed will be key to implementing the trials. More experienced researchers may be required?<br>• No overhead budgets are present fro the Canadian Universities? This should be clarified. | 4 |
| **Evaluator 2** | The work packages for VoiceAdapt are well constructed and appropriate for the proposal's objectives with a good balance of expertise and involvement. Risk assessment has been considered and although the target number of users involved in trials is modest, the budgets seem appropriate for the tasks involved | 5 |
| **Evaluator 3** | Good design seems to be doable | 3 |

| **5. Potential impact on society and policy** | Response to actual societal needs, providing evidence for policy makers and practitioners; early integration of relevant stakeholders, ensuring societal relevance over the course of the project and its dissemination | **Score** |
|---|---|---|
| **Evaluator 1** | VoiceAdapt presents a clear proposition to empower older adults with aphasia, by providing them with an autonomously usable low cost solution. Improving the language capabilities of individuals significantly affected by Stroke is a major challenge and highly important to improving their overall quality of life is the ability to effectively participate in rehabilitation. The intelligent personalisation model outlined in this proposal has the potential | 5 |

| | | |
|---|---|---|
| | to significantly enhance the experience of those living with aphasia post stroke.<br><br>Clear and strong added value is presented throughout the proposal to the research field and with regards societal impact.<br><br>The main RCT will occur in Canada, a more detailed understanding of how the outcomes will impact back on the EU should be presented.<br><br>Project impact is comprehensively covered. The consortium outlines impacts that will lead to the creation of new products and services around supportive speech training systems, however the underlying impact from this programme will be to enhance patient, health professional and speech and language therapist engagement with an adaptive system that can target individuals less able to make hospital visits. The ability for VoiceAdapt to enhance remote rehabilitation for individuals with aphasia is substantial. A further impact will lie in the cost savings to health services (and wider society) able to provide such a system, given the high prevalence of stroke, particularly with an increasingly younger population.<br><br>P9 - Proposal states that solution impact on patient empowerment is measured across the various domains of the WHO-ICF framework. This should be embellished to understand exactly how this will occur in the project.<br><br>A clear and well presented approach to dissemination across academic, industry and public interests is presented, which takes into consideration the national states of consortium partners, wider EU and America.<br><br>The proposal provides a brief approach to the exploitation strategy, mapped against the business model canvas with well specified dissemination and exploitation plans for each partner specifically. | |
| **Evaluator 2** | The growing percentage of elderly people, extended life expectancies and the prevalence of lifestyle related medical conditions provides evidence of the value of solutions that support self-management of chronic conditions with links to medical practitioners for remote support. There is strong evidence of target user engagement and the results of the project will inform both policy makers and practitioners. The impact on society is only limited by the number of people suffering from Aphasia now and in the future. | 4 |
| **Evaluator 3** | If scuccessful the tool can be of great importance to enhance quality of Life for people living with aphasia. | 4 |

| **6. Gender dimension** | A proposal is considered gender relevant when it can be expected that its findings affect women and men or groups of women and men differently. In these cases, applicants should integrate gender issues and, when relevant specific studies, as part of the proposals. Gender balance in applicants' consortia will be noted | **Score** |
|---|---|---|
| **Evaluator 1** | An adequate breakdown of gender issues facing both the research and team structure is presented. Some specifity around the existing methods and approaches they will use should be provided. | 3 |
| **Evaluator 2** | The proposal recognises the relevance of gender in the incidence of Aphasia and addresses the issue adequately. The consortium already has a good gender balance. | 5 |
| | Good and relevant | 4 |

| | | |
|---|---|---|
| **Evaluator 3** | | |
